# Complement C3 serum levels in anorexia nervosa: a potential biomarker for the severity of disease?

**DOI:** 10.1186/1744-859X-10-16

**Published:** 2011-05-04

**Authors:** Michael A Flierl, Jennifer L Gaudiani, Allison L Sabel, Carlin S Long, Philip F Stahel, Philip S Mehler

**Affiliations:** 1 Department of Orthopedics, Denver Health Medical Center, Denver, CO, USA; 2 Department of Internal Medicine, Denver Health Medical Center, Denver, CO, USA; 3 Department of Patient Safety and Quality, Denver Health Medical Center, Denver, CO, USA; 4 Department of Biostatistics and Informatics, Denver Health Medical Center, Denver, CO, USA; 5 Division of Cardiology, Department of Medicine, Denver Health Medical Center, Denver, CO, USA; 6 Department of Neurosurgery, University of Colorado Denver, School of Medicine, Denver, CO, USA

## Abstract

**Background:**

Anorexia nervosa carries the highest mortality rate of any psychiatric disorder. Even the most critically ill anorexic patients may present with normal 'standard' laboratory values, underscoring the need for a new sensitive biomarker. The complement cascade, a major component of innate immunity, represents a driving force in the pathophysiology of multiple inflammatory disorders. The role of complement in anorexia nervosa remains poorly understood. The present study was designed to evaluate the role of complement C3 levels, the extent of complement activation and of complement hemolytic activity in serum, as potential new biomarkers for the severity of anorexia nervosa.

**Patients and methods:**

This was a prospective cohort study on 14 patients with severe anorexia nervosa, as defined by a body mass index (BMI) <14 kg/m^2^. Serum samples were obtained in a biweekly manner until hospital discharge. A total of 17 healthy subjects with normal BMI values served as controls. The serum levels of complement C3, C3a, C5a, sC5b-9, and of the 50% hemolytic complement activity (CH50) were quantified and correlated with the BMIs of patients and control subjects.

**Results:**

Serum C3 levels were significantly lower in patients with anorexia nervosa than in controls (median 3.7 (interquartile range (IQR) 2.5-4.9) vs 11.4 (IQR 8.9-13.7, *P *<0.001). In contrast, complement activation fragments and CH50 levels were not significantly different between the two groups. There was a strong correlation between index C3 levels and BMI (Spearman correlation coefficient = 0.71, *P *<0.001).

**Conclusions:**

Complement C3 serum levels may represent a sensitive new biomarker for monitoring the severity of disease in anorexia nervosa. The finding from this preliminary pilot study will require further investigation in future prospective large-scale multicenter trials.

## Introduction

Anorexia nervosa occurs in an estimated 0.9% of women and 0.3% of men in the US alone [1]. The treatment course is usually lengthy and challenging due to potentially life-threatening medical complications that can affect almost every organ system [2]. Such impediments result in the highest death rates (approximately 5%) of any psychiatric disorder [2,3]. In fact, the overall mortality rate in anorexia nervosa patients is about 10 times higher than the expected mortality for age-matched women in the US [2,3]. Published guidelines support hospitalization for medical stabilization when patients with anorexia nervosa weigh less than 70% of their calculated ideal body weight (IBW), have severe bradycardia (≤50 beats/min), severe hypotension, or life threatening electrolyte abnormalities [2,4,5]. In this population of young, usually otherwise healthy patients with pure food restriction, normal serum albumin levels frequently mask the severity of their serious medical condition [6,7]. A recent analysis of patients with severe anorexia nervosa (median body mass index 13.1 kg/m^2^) admitted for medical stabilization showed that most patients, despite profoundly low body weight, have normal laboratory values on admission, with the exception of lymphopenia and anemia due to starvation-mediated bone marrow suppression [2,4]. However over the course of the early weeks of refeeding, nearly half developed hypoglycemia, three-quarters showed abnormal liver function most likely related to starvation-induced autophagy, 83% showed abnormal bone density, nearly half developed refeeding hypophosphatemia, and 92% were hypothermic [8,9]. Despite the significant abnormalities and the extent of bone marrow suppression, these critically ill patients do not manifest significant inflammatory or infectious processes. Outcome measures of medical stability draw from a combination of factors including improvement of standard laboratory values, ingestion of adequate calories to begin weight restoration, and resolution of comorbidities [4,10,11]. To date, no appropriate biomarker exists to monitor treatment success or progression of disease [4,12].

Complement represents one of the phylogenetically oldest cascade systems. As an important effector of the innate immune response, the complement system represents the 'first line of defense' against invading pathogens [13]. Although of beneficial intention, excessive complement activation has been associated with detrimental effects related to 'innocent bystander' host cell injury [14]. Disproportionate complement activation in sepsis, for example, appears to play a key role in the pathophysiology of neutrophil dysfunction, coagulopathy, apoptotic events and cardiomyopathy [15,16]. There is relative paucity of data on the role of complement proteins in anorexia nervosa in the literature and no correlations were made between complement levels and the severity of disease. Moreover, anorexia nervosa has traditionally been viewed as an illness with malnourishment, but devoid of a prominent inflammatory component, as shown by surprisingly normal albumin levels [6,7].

The present study was designed to assess complement activation in patients with severe anorexia nervosa, and to determine whether complement serum levels may represent a useful marker for determining and monitoring the severity of disease. We hypothesized that anorexia nervosa results in complement activation and consumption of complement C3, the central component of all complement activation pathways.

## Patients and methods

### Setting

The Acute Comprehensive Urgent Treatment of Eating disorders (ACUTE) center at Denver Health Medical Center is a five-bed, multidisciplinary center that cares for the largest number of critically ill anorexic patients in the country. It serves patients too medically compromised to initiate or continue treatment in a psychiatrically based eating disorder program. Therefore, the ACUTE center is a medical stabilization unit treating the most seriously ill anorexic patients. Although hospitalization is recommended for anorexic patients with a body mass index (BMI) under 14 kg/m^2^, the ACUTE center's patients have a mean BMI of 12.6 kg/m^2^, making them a uniquely ill patient population. It is worth noting that the *Diagnostic and Statistical Manual of Mental Disorders*, fourth edition (DSM-IV) criteria for anorexia nervosa defines this illness as having a BMI <17.5. Thus the patients in this study have extremely severe forms of anorexia nervosa. Upon primary medical stabilization, patients are transferred to a psychiatrically based inpatient eating disorder program further treatment and follow-up.

### Patients and controls

The present study was designed as a prospective cohort study. Prior to study initiation, approval by the Institutional Review Board was obtained. Patients admitted to the ACUTE center with a diagnosis of severe anorexia nervosa were consented and enrolled into the study (n = 14). Blood samples were obtained every 2 weeks and demographic data, routine laboratory parameters (complete blood counts, electrolytes and minerals, hepatic function tests), and body weight were assessed in standard fashion until discharge. Patients provided blood samples only during their hospitalization so patients did not contribute the same number of samples to the study. A healthy volunteer group of 17 individuals served as the control group for this study and blood samples were obtained once.

### Blood sampling

Whole blood was sampled via venipuncture of the anticubital vein. Serum tubes were used in all cases, and were immediately put on ice. Serum was collected after clotting and centrifugation at 800 *g *for 10 min at 4°C. To avoid repeated freeze-thaw cycles, samples were aliquoted and stored at -80°C until further analysis.

### Protein measurements

The total protein content of serum samples was quantified using BCA protein measurement (Thermo Scientific, Rockford, IL, USA) according to the manufacturer's instructions. Samples were diluted 1:100 in phosphate-buffered saline (PBS) prior to incubation and spectrophotometric assessment. Bovine serum albumin (Thermo Scientific) was used to generate a standard curve.

### Complement hemolytic activity and serum levels

Serum samples were thawed and processed immediately. Repetitive freeze-thaw cycles were avoided to minimize *in vitro *complement activation. The following commercially available ELISA kits were used strictly according to the manufacturer's protocol: MicroVue CH50 Eq EIA kit (Quidel, San Diego, CA, USA; sample dilution per manufacturer's protocol); C3 fixed complement preceptor ELISA kit (Bachem, San Carlos, CA, USA; 1:20 sample dilution); MicroVue C3a EIA kit (Quidel; 1:150 sample dilution); human complement component C5a ELISA (R&D, Minneapolis, MN, USA; 1:20 sample dilution); MicroVue sC5b-9 EIA kit (Quidel). Obtained concentrations were protein adjusted (concentration/mg total protein) in order to address differences in total protein levels between anorexia nervosa patients and healthy volunteers.

### Statistical analysis

Baseline characteristics of the study participants are described with mean and standard deviation (SD) or percentages. Complement levels are expressed as medians with interquartile ranges because they were non-normally distributed (Anderson-Darling test). Comparisons between the anorexia nervosa patients and healthy volunteers were analyzed with an unpaired t test, Wilcoxon rank sum test, or Fisher's exact test, as appropriate. Spearman correlation coefficients were used to determine association between BMI and complements. A generalized estimating equation (GEE) analysis was used to determine the relationship between BMI and complement level over time for the anorexia nervosa patients. PROC GENMOD was used since it accounts for the repeated measures within a patient, allows missing data, and does not require the response to be normally distributed. Differences were considered significant when *P *<0.05. All analyses were conducted in SAS v.9.1. (SAS, Cary, NC, USA).

## Results

### Patient demographics

The study consisted of two cohorts. In all, 14 anorexia nervosa patients were compared with 17 healthy controls (Table 1). In the anorexia group, 79% of the patients were women, with a mean age of 32.4 years (SD 12.8). The healthy controls had similar characteristics. At the time of admission, the anorexia group had a mean initial body mass index of 13.6 ± 1.5 kg/m^2 ^compared with 22.2 ± 2.6 kg/m^2 ^in the control group (*P *<0.001). The initial percentage of ideal body weight in the anorexia group was 64.5 ± 7.6%, which was 40% lower than the healthy controls (*P *<0.001). The serum total protein was 61.9 ± 8.2 mg/ml in the anorexia group compared with 73.7 ± 9.6 in the control group, *P *= 0.001. Thus, we adjusted for this baseline difference in protein when comparing complement activation between the two groups. The mean admission albumin level for the anorexia nervosa patients was within normal limits (3.6 ± 0.7 g/dl, normal range 3-5.3 g/dl). Anorexic patients were followed for a mean of 43 ± 8.8 days. No patient had evidence of systemic infection, malignancy, shock, vascular disease, or rheumatologic disease. The peripheral blood cell counts in the anorexia cohort are shown in Table 2.

**Table 1:** Patient demographics

Demographic	Healthy controls	Anorexia nervosa patients	*P *value^a^
Patients, n	17	14	-

Age, years	30.4 ± 3.7	32.4 ± 12.8	0.56

Female, %	82.4	78.6	>0.99

Height, inches	66.4 ± 4.4	64.0 ± 3.2	0.09

Initial weight, pounds	140.6 ± 31.4	80.6 ± 11.6	<0.001

Initial body mass index, kg/m^2^	22.2 ± 2.6	13.6 ± 1.5	<0.001

Initial percentage of ideal body weight	104.1 ± 11.4	64.5 ± 7.6	<0.001

Serum total protein, mg/ml	73.7 ± 9.6	61.9 ± 8.2	0.001

Admission albumin levels (normal: 3-5.3 g/dl)	NA	3.6 ± 0.7	NA

Length of follow-up, days	NA	49.4 ± 31.7^b^	NA

^a^Statistical analysis compares differences between cohorts using t test and Fisher's exact test as appropriate. Data are expressed as mean ± SD.

^b^Four anorexia patients were in the hospital less than 2 weeks; therefore they did not have a follow-up and are excluded.

NA = not applicable.

**Table 2:** Peripheral blood cell count in patients with anorexia nervosa (n = 14).

Cell count (laboratory normal range)	Mean ± SD or median (IQR)	Range
Neutrophils (48.0% to 69.0%)	56.2% ± 11.3%	37.9% to 77.5%

Absolute neutrophils (2.0-7.0 k/μl)	2.1 (1.4-3.4)	1.2-6.9

Lymphocytes (21.0%-43.0%)	33.6% ± 10.9%	16.7% to 53.0%

Absolute lymphocytes (0.9-4.0 k/μl)	1.4 ± 0.5	0.6-2.4

### Complement hemolytic activity and serum levels

**Figure 1:**
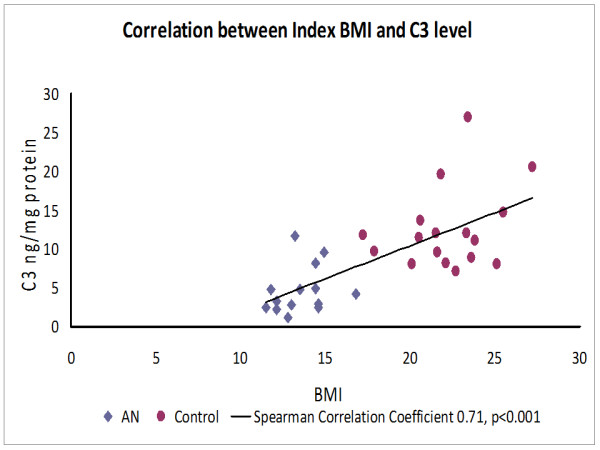
**Correlation between body mass index (BMI) and complement C3 serum levels in patients with anorexia nervosa (AN) and healthy controls**.

Complement activation was determined in serum samples from anorexia nervosa patients (n = 14) obtained on admission and compared to the healthy control cohort (n = 17). Complement analysis is depicted in Table 3. Serum levels of C3 were threefold lower in patients with anorexia nervosa than in controls (median 3.7 (interquartile range (IQR) 2.5-4.9) vs 11.4 (8.9-13.7), *P *<0.001). The serum levels of complement activation fragments (C3a, C5a, C5b-9) and the extent of 50% hemolytic complement activity (CH50) were not significantly different between the two groups. In contrast, C3 levels were strongly correlated with index BMI (Spearman correlation coefficient = 0.71, *P *<0.001; Figure 1). Complement C3 levels did not correlate with BMI in the anorexia group alone (n = 14, Spearman correlation coefficient = 0.36, *P *= 0.20).

**Table 3:** Complement levels in anorexia patients and controls

Complement activation adjusted for protein	Healthy controls	Anorexia nervosa patients (admission laboratory test results)	*P *value^a^
Patient numbers, n	17	14	

CH50, U Eq/ml	1.8 (1.4-2.2)	1.6 (0.9-2.0)	0.64

**C3, ng/mg protein**	**11.4 (8.9-13.7)**	**3.7 (2.5-4.9)**	**<0.001**

C3a, ng/mg protein	1,985 (1,817-2,488)	1,799 (1,295-3,752)	0.83

C5a, pg/mg protein	437 (412-477)	469 (439-537)	0.10

sC5b-9, ng/mg protein	1.9 (0.7-4.6)	2.8 (2.0-6.8)	0.22

^a^Concentrations were assessed in serum samples. Statistical analysis compares concentrations between cohorts using Wilcoxon rank sum test. Data are expressed as median (interquartile range).

^b^Laboratory samples were unavailable for one patient for C5a and CH50, nine controls for CH50, and two controls for sC5b-9.

CH50 = 50% hemolytic complement activity.

### Increase in BMI correlates with complement activation

Serum samples were obtained from anorexia nervosa patients in a biweekly manner after initiation of refeeding and analyzed for levels of CH50, C3, C3a, C5a and sC5b-9. Each patient provided between one and six sets of laboratory results depending on the length of their hospitalization. Eight patients (57%) had multiple blood samples and were included in the longitudinal analysis. As the patients became medically stabilized during their admission, BMI increases over time were not statistically correlated with C3 changes.

## Discussion

This study provides first evidence of significantly decreased complement C3 levels in patients with severe anorexia nervosa, compared to healthy control subjects. There was a strong correlation between index C3 levels and patients' and controls' BMI values (Spearman correlation coefficient = 0.71, *P *<0.001), suggesting that serum C3 levels may represent a clinically relevant serum marker reflecting the severity of disease, and potentially serving as a guide for monitoring the refeeding process. That is, in patients with anorexia nervosa and severely low body weight, in whom basic laboratory tests are often normal, low serum C3 levels can confirm biochemical evidence of severe illness. It is reasonable that serum C3 levels did not correlate with BMI in the anorexia group alone, reflective of the fact that a 'threshold' of severe illness from anorexia has been crossed at these profoundly low body weights, which occasioned the low C3 level. The fact that serum C3 levels did not statistically correlate with weight restoration over the course of treatment may have a complex explanation, and thus restoration of non-edematous weight remains the best marker of physiologic recovery in anorexia nervosa. In contrast to the findings on C3 concentrations, serum levels of complement activation fragments (C3a, C5a, C5b-9) and the extent of complement hemolytic activity (CH50) did not significantly correlate with the patients' BMIs. There are several potential explanations for this negative finding. First, a recent study described direct cleavage of C5 via thrombin, thereby bypassing the traditional activation cascade using C3 convertases or C5 convertases [13]. As a result, C5a may be generated via thrombin-mediated coagulation abnormalities that have been documented in anorexia nervosa patients [17,18]. In addition, phacocytic cells are able to directly cleave C5 and locally generate C5a [19]. Maj and colleagues revealed that peripheral mononuclear cells (PBMCs) isolated from anorexia nervosa patients exhibited significantly elevated levels of activated intracellular G proteins, indicating increased PBMC activity in these patients [20]. Thus, activated PBMCs and neutrophils may further contribute to alterations of C3a and C5a levels bypassing the traditional complement activation cascade.

There are few reports on complement activation in anorexia nervosa available in the peer-reviewed literature, dating back to the 1970s and 1980s [21-23]. Wyatt *et al*. published a series of five anorexia nervosa patients and observed significantly decreased serum levels of C1q, C2, C3, factor B, β leutenizing hormone (β-LH), C3b inactivator, properdin, and C4 binding protein [22]. After initiation of alimentation, β-LH, C3b inactivator, C3, and factor B rapidly returned to the normal range in response to therapy [22]. In line with these findings, Sigal and colleagues found low serum levels of complement proteins in anorexia nervosa patients [23]. A more recent report evaluated several components of the complement cascade and analyzed the activities of the alternative complement activation pathways [24]. Serum levels of C3, Factor B and D, hemolytic activity of the alternative pathway, and the inhibitors H and I were found to be low in anorexia patients and normalized with weight gain [24]. In our current study, we determined low C3 levels in anorexic patients, which is in line with those previous findings. However, while our findings suggest complement consumption secondary to increased activation in anorexic patients, Pomeroy and colleagues concluded that low serum complement levels were attributable to hypoproduction as opposed to increased consumption, and that percentage of ideal body weight, changes in body weight, and serum transferrin were each highly correlated with serum levels of complement proteins [24]. These differences to our findings may be due to the fact that Pomeroy and colleagues assessed functional capacity of the alternative complement activation pathway exclusively, while our study focused on complement activation via the classical pathway (CH50) and complement activity further downstream (C3a, C5a, MAC). Moreover, Pomeroy *et al*. failed to adjust their samples to total protein levels, which may have resulted in variable protein concentrations. In the present study, anorexia nervosa patients had significantly lower serum total protein levels than healthy controls on admission (61.9 ± 2.2 mg/ml vs 73.7 ± 2.3 mg/ml; *P *= 0.003) [24]. Nova and colleagues evaluated several biochemical markers in 14 anorexia nervosa patients and compared them to a healthy control cohort of (n = 15) [25]. The authors reported significantly increased concentrations of C3 (and C4) upon admission in anorexia nervosa patients [25]. At the 1-year follow-up, C3 and C4 levels had returned to levels comparable to the healthy control cohort. Nova *et al*. also failed to adjust their measurements to total protein levels in their samples, which may account for the differences observed.

Our study has several limitations. First, the low patient numbers limit the power of our statistical analysis and make our data vulnerable to a statistical type II error. Therefore, our data do not allow for advocating complement serum levels as a new biomarker until definitively proven in future large-scale prospective studies. Moreover, follow-up studies will have to determine during which time frame complement levels return to healthy control levels after initiation of refeeding protocols, and whether complement serum levels may represent a valuable tool to monitor therapy success or failure in anorexia patients. Nevertheless, to our knowledge, our study is the first to describe a full complement screening in severely ill anorexia nervosa patients upon admission, and to correlate complement levels with gain of body weight as a function of time.

## Conclusions

The complement system represents a crucial effector of the acute phase response of innate immunity. Excessive complement activation, however, has been implicated in the pathophysiology of various inflammatory diseases [14,16,26]. Therefore, it is conceivable that the increased complement activation observed in anorexia nervosa patients may be involved in the development of complications associated with severe anorexia nervosa. The pharmacological complement blockade has been shown to ameliorate the severity of numerous diseases, including sepsis [27], neuroinflammation [14,28], chest trauma [29], and ischemia reperfusion injury [30]. Therefore, it appears reasonable to hypothesize that the pharmacological blockade of the complement cascade or complement receptors may represent a future therapeutic treatment strategy to reduce the incidence of anorexia nervosa-associated complications. In conclusion, future prospective large-scale studies will have to determine the value of complement serum levels as potential biomarkers to monitor treatment success or failure in patients with severe anorexia nervosa.

## Competing interests

The authors declare that they have no competing interests.

## Authors' contributions

MAF, JLG, PFS and PSM designed the study. MAF performed the sample analysis and the statistical evaluation. JLG consented the patients and reviewed the demographic data. MAF, JLG, PFS and PSM wrote the manuscript. CSL reviewed the manuscript. All authors contributed to the revisions of the text and approved the final version of this manuscript.

## References

[B1] Hudson JI Hiripi E Pope HG Jr Kessler RC The prevalence and correlates of eating disorders in the National Comorbidity Survey Replication Biol Psychiatry 2007 61 348 358 10.1016/j.biopsych.2006.03.040 16815322 PMC1892232

[B2] Mehler PS Krantz M Anorexia nervosa medical issues J Womens Health 2003 12 331 340 10.1089/154099903765448844 12804340

[B3] Steinhausen HC The outcome of anorexia nervosa in the 20th century Am J Psychiatry 2002 159 1284 1293 10.1176/appi.ajp.159.8.1284 12153817

[B4] Mehler PS Winkleman AB Andersen DM Gaudiani JL Nutritional rehabilitation: practical guidelines for refeeding the anorectic patient J Nutr Metab 2010 pii 625782 10.1155/2010/625782 PMC2925090 20798756

[B5] Schwartz BI Mansbach JM Marion JG Katzman DK Forman SF Variations in admission practices for adolescents with anorexia nervosa: a North American sample J Adolesc Health 2008 43 425 431 10.1016/j.jadohealth.2008.04.010 18848669

[B6] Krantz MJ Lee D Donahoo WT Mehler PS The paradox of normal serum albumin in anorexia nervosa: a case report Int J Eat Disord 2005 37 278 280 10.1002/eat.20129 15822081

[B7] Narayanan V Gaudiani JL Mehler PS Serum albumin levels may not correlate with weight status in severe anorexia nervosa Eat Disord 2009 17 322 326 10.1080/10640260902991202 19548148

[B8] Gaudiani J Sabel AL Mascolo M Mehler PS Severe anorexia nervosa: Outcomes from a medical stabilization unit Int J Eat Disord in press 10.1002/eat.20889 22170021

[B9] Mehler PS Weiner KL Use of total parenteral nutrition in the refeeding of selected patients with severe anorexia nervosa Int J Eat Disord 2007 40 285 287 10.1002/eat.20371 17262814

[B10] Narayanan V Gaudiani J Harris RH Mehler PS Liver function test abnormalities in anorexia nervosa - cause or effect Int J Eat Disord 2010 43 378 381 19424979 10.1002/eat.20690

[B11] Mehler PS MacKenzie TD Treatment of osteopenia and osteoporosis in anrexia nervosa: a systematic review of the literature Int J Eat Disord 2009 42 195 201 10.1002/eat.20593 18951456

[B12] Prince AC Brooks SJ Stahl D Treasure J Systematic review and meta-analysis of the baseline concentrations and physiologic responses of gut hormones to food in eating disorders Am J Clin Nutr 2009 89 755 765 10.3945/ajcn.2008.27056 19176730

[B13] Huber-Lang M Sarma JV Zetoune FS Rittirsch D Neff TA McGuire SR Lambris JD Warner RL Flierl MA Hoesel LM Gebhard F Younger JG Drouin SM Wetsel RA Ward PA Generation of C5a in the absence of C3: a new complement activation pathway Nat Med 2006 12 682 687 10.1038/nm1419 16715088

[B14] Stahel PF Barnum SR The role of the complement system in CNS inflammatory diseases Expert Rev Clin Immunol 2006 2 445 456 10.1586/1744666X.2.3.445 20476915

[B15] Rittirsch D Flierl MA Ward PA Harmful molecular mechanisms in sepsis Nat Rev Immunol 2008 8 776 787 10.1038/nri2402 18802444 PMC2786961

[B16] Schreiber H Rittirsch D Flierl M Brueckner U Schneider M Weiss M Gebhard F Huber-Lang M Complement activation during sepsis in humans Adv Exp Med Biol 2006 586 217 226 10.1007/0-387-34134-X_15 16893075

[B17] Gaudiani JL Kashuk JL Chu ES Narayanan V Mehler PS The use of thrombelastography to determine coagulation status in severe anorexia nervosa: a case series Int J Eat Disord 2010 43 382 385 19418569 10.1002/eat.20691

[B18] Cleary BS Gaudiani JL Mehler PS Interpreting the complete blood count in anorexia nervosa Eat Disord 2010 18 132 139 10.1080/10640260903585540 20390616

[B19] Huber-Lang M Younkin EM Sarma JV Riedemann N McGuire SR Lu KT Kunkel R Younger JG Zetoune FS Ward PA Generation of C5a by phagocytic cells Am J Pathol 2002 161 1849 1859 10.1016/S0002-9440(10)64461-6 12414531 PMC1850785

[B20] Monteleone P Di Lieto A Martiadis V Pannuto M Maj M Altered immunoreactive levels of G proteins in peripheral mononuclear cells of patients with anorexia nervosa and bulimia nervosa Mol Psychiatry 2003 8 680 684 10.1038/sj.mp.4001304 12874604

[B21] Palmblad J Fohlin L Norberg R Plasma levels of complement factors 3 and 4, orosomucoid and opsonic functions in anorexia nervosa Acta Paediatr Scand 1979 68 617 618 10.1111/j.1651-2227.1979.tb05067.x 463547

[B22] Wyatt RJ Farrell M Berry PL Forristal J Maloney MJ West CD Reduced alternative complement pathway control protein levels in anorexia nervosa: response to parenteral alimentation Am J Clin Nutr 1982 35 973 980 6805290 10.1093/ajcn/35.5.973

[B23] Sigal LH Snyder BK Low serum complement levels in anorexia nervosa Am J Dis Child 1989 143 1391 1392 2589267 10.1001/archpedi.1989.02150240013004

[B24] Pomeroy C Mitchell J Eckert E Raymond N Crosby R Dalmasso AP Effect of body weight and caloric restriction on serum complement proteins, including Factor D/adipsin: studies in anorexia nervosa and obesity Clin Exp Immunol 1997 108 507 515 10.1046/j.1365-2249.1997.3921287.x 9182900 PMC1904692

[B25] Nova E Lopez-Vidriero I Varela P Toro O Casas JJ Marcos AA Indicators of nutritional status in restricting-type anorexia nervosa patients: a 1-year follow-up study Clin Nutr 2004 23 1353 1359 15556257 10.1016/j.clnu.2004.05.004

[B26] Flierl MA Stahel PF Rittirsch D Huber-Lang M Niederbichler AD Hoesel LM Touban BM Morgan SJ Smith WR Ward PA Ipaktchi K Inhibition of complement C5a prevents breakdown of the blood-brain barrier and pituitary dysfunction in experimental sepsis Crit Care 2009 13 R12 10.1186/cc7710 19196477 PMC2688129

[B27] Czermak BJ Sarma V Pierson CL Warner RL Huber-Lang M Bless NM Schmal H Friedl HP Ward PA Protective effects of C5a blockade in sepsis Nat Med 1999 5 788 792 10.1038/10512 10395324

[B28] Leinhase I Schmidt OI Thurman JM Hossini AM Rozanski M Taha ME Scheffler A John T Smith WR Holers VM Stahel PF Pharmacological complement inhibition at the C3 convertase level promotes neuronal survival, neuroprotective intracerebral gene expression, and neurological outcome after traumatic brain injury Exp Neurol 2006 199 454 464 10.1016/j.expneurol.2006.01.033 16545803

[B29] Flierl MA Perl M Rittirsch D Bartl C Schreiber H Fleig V Schlaf G Liener U Brueckner UB Gebhard F Huber-Lang MS The role of C5a in the innate immune response after experimental blunt chest trauma Shock 2008 29 25 31 17621257 10.1097/shk.0b013e3180556a0b

[B30] Riedemann NC Ward PA Complement in ischemia reperfusion injury Am J Pathol 2003 162 363 367 10.1016/S0002-9440(10)63830-8 12547694 PMC1851148

